# First-Line Pharmacotherapies and Survival among Patients Diagnosed with Non-Resectable NSCLC: A Real-Life Setting Study with Gender Prospective

**DOI:** 10.3390/cancers13236129

**Published:** 2021-12-05

**Authors:** Andrea Spini, Rosa Gini, Pietro Rosellini, Allison Singier, Cristiana Bellan, Alessandra Pascucci, Lorenzo Leoncini, Clément Mathieu, Ignazio Martellucci, Folco Furiesi, Silvano Giorgi, Sandra Donnini, Giuseppe Roberto, Marina Ziche, Francesco Salvo

**Affiliations:** 1 INSERM, BPH, U1219, Team Pharmacoepidemiology, Université de Bordeaux, 33000 Bordeaux, France; allison.singier@u-bordeaux.fr (A.S.); clement.mathieu@u-bordeaux.fr (C.M.); 2 Department of Medical Science, Surgery and Neuroscience, University of Siena, 53100 Siena, Italy; ziche@unisi.it; 3 Osservatorio di Epidemiologia, Agenzia Regionale di Sanità Toscana, 50141 Florence, Italy; rosa.gini@ars.toscana.it (R.G.); giuseppe.roberto@ars.toscana.it (G.R.); 4 CIC 1401, CIC Bordeaux, 33000 Bordeaux, France; pietro.rosellini@u-bordeaux.fr; 5 Pole de Santé Publique, Service de Pharmacologie Médicale, Centre de Pharmacovigilance de Bordeaux, CHU de Bordeaux, 33000 Bordeaux, France; 6 Department of Medical Biotechnology, University of Siena, 53100 Siena, Italy; cristiana.bellan@unisi.it (C.B.); lorenzo.leoncini@unisi.it (L.L.); 7 Azienda Ospedaliera Universitaria Senese, 53100 Siena, Italy; ale.pascucci@ao-siena.toscana.it (A.P.); igmartellucci@gmail.com (I.M.); f.furiesi@estar.toscana.it (F.F.); 8 Azienda USL Toscana Sud Est, 53100 Siena, Italy; silvano.giorgi@uslsudest.Toscana.it; 9 Department of Life Sciences, University of Siena, 53100 Siena, Italy; sandra.donnini@unisi.it

**Keywords:** drug utilization, immunotherapy, NSCLC, non-small cell lung cancer, target therapy, survival, gender differences, observational study

## Abstract

**Simple Summary:**

Women and men have a different biomolecular profile that could impact drug utilization and survival in non-small cell lung cancer (NSCLC) patients. The aim of the study was to describe first-line pharmacotherapy and overall survival in non-resectable (nr)NSCLC patients by gender. About 4400 incident cases of nrNSCLC were included. We reported a different use of target therapies on the basis of the known biomolecular profile between the two sexes. The survival improved in the last decade, and women and men also showed different survival if diagnosed with a squamous or non-squamous nrNSCLC.

**Abstract:**

(1) Purpose: To describe first-line pharmacotherapy and overall survival in non-resectable non-small cell lung cancer (nrNSCLC) patients by gender. (2) Methods: Incident cases of nrNSCLC recorded between 2009 and 2019 (cohort entry) in the pathology registry of the regional administrative healthcare database of Tuscany were identified. Records of antineoplastic therapies delivered up to 4 months following cohort entry were classified as chemotherapy, target therapies, immunotherapies, and undefined monoclonal antibodies. First-line treatment and survival of patients receiving drug treatment was described. Analyses were stratified according to histology, gender, and cohort entry year. (3) Results: 4393 incident cases of nrNSCLC were included. Women with non-squamous-NSCLC received target-therapy more frequently than men (14.9% vs. 6.5%). Immunotherapy incidence of use varied between 3.8% (2017) and 9.1% (2019). The 2-year survival rate increased over time: for non-squamous-NSCLC, it was 22.3% (2009–2011) and 30.6% (2018–2019), while for squamous-NSCLC, it was 13.5% and 22.5%, respectively. After multivariate analysis, a low reduction in mortality risk in 2018–2019 vs. 2009–2011 was found (non-squamous: HR: 0.95 CI95%: 0.92–0.98; squamous: HR: 0.94 CI95%: 0.90–0.98). Among non-squamous NSCLC, median survival was longer in women than in men (389 vs. 276 days). (4) Conclusion: In light of sex-related biomolecular differences, among non-squamous NSCLC, women received target-therapy more frequently than men. Survival seemed to slightly improve over the study period for both histologies, despite a poor reduction in mortality risk was still observed.

## 1. Introduction

Lung cancer is the most commonly diagnosed cancer in the world. It represents 14.5% of total cancer cases in men and 8.4% in women, being the leading cause of cancer death in men (22.0%). As a result of the reduction in the smoking habit in men and the increase in women, in recent years, there has been a marked reduction in the incidence of this cancer in men, amounting to −1.6% per year, while in women, there has been an average increase of 1.7% per year [1,2].

Based on histological characteristics, the World Health Organization divides lung cancer into two classes: small cell lung cancer and non-small cell lung cancer (NSCLC). The latter represents 85% of all cases of lung cancer, and its diagnosis is usually based on diagnostic imaging (chest X-ray and computed tomography) and the anatomopathological evaluation of a biological sample [2].

The two main histological subtypes of NSCLC are: squamous and non-squamous carcinoma (mainly adenocarcinoma and large cell carcinoma). According to epidemiological data, the latter subtype is proportionally much more frequent in women than in men [3]. Regardless of histological type, in the early stages of the disease, the NSCLC shows no obvious clinical symptoms, and the first-choice treatment is the surgical resection of the neoplastic tissue. However, it often happens that the diagnosis of NSCLC is performed when the tumor is already advanced or metastatic and therefore no longer eligible for surgical resection, and so drug treatment becomes the first therapeutic choice.

During the last decade, the pharmacological treatment of non-resectable NSCLC has undergone a real revolution thanks to the licensing of different innovative antineoplastic medications, namely target therapy and immunotherapy drugs [2]. Table S1 in Supplementary Materials shows all the target and immuno-therapies approved for advanced/metastatic NSCLC, with the related approval date by EMA. The first anti-Epithelia growth factor receptor (*EGFR*) approved as first line was gefitinib, in 2009 by EMA, followed by Crizotinib, an Anaplastic lymphoma kinase (*ALK*) inhibitor, in 2012. More recently, immunotherapies such as nivolumab (2015) were also approved by EMA as second/third line treatment for advanced-stage NSCLC, while in 2016, the use of pembrolizumab as a first-line treatment was also approved.

Today, real-world evidence on the utilization of innovative anticancer treatments for NSCLC is still poor, particularly in Europe [4]. In this respect, existing electronic healthcare databases that routinely collect information from everyday clinical practice have tremendous potential for filling such gaps of knowledge. Moreover, the importance of sex and gender as modulator of treatment use and outcomes in cancer patients is still an under-evaluated issue, and the reporting of results by sex in publications need to be reemphasized in oncology [5]. These sources of observational data can be re-used to execute large pharmaco-epidemiological studies to generate evidence on the utilization of anti-cancer medications in a real-word setting by gender [6,7].

Over the last two decades, in Tuscany region, in Italy, a region-wide population-based administrative healthcare database has collected information on healthcare services reimbursed by the National Health Service and delivered to Tuscan inhabitants (around 3.7 million subjects). Although the administrative healthcare database of Tuscany has been extensively used for pharmaco-epidemiological studies [8,9,10], no experience currently exists in linking patient-level data from the different available registries to the information recorded in the regional pathology registry to describe the utilization of anticancer medications.

The aim of this study was to describe survival and drug utilization of first-line anticancer therapies approved for the treatment of advanced, non-resectable, NSCLC patients in relation to gender by analyzing data from the regional administrative database of Tuscany.

## 2. Materials and Methods

### 2.1. Data Sources

Data from the regional administrative database of Tuscany region were used. The database collects longitudinal pseudonymized patient-level information on the utilization of healthcare services reimbursed by the National Healthcare Service and dispensed to all Tuscan inhabitants who are registered with a general practitioner. For each subject registered in the inhabitant registry of the database, demographic data and vital status can be linked to different registries. In particular, the regional pathology registry was used for the identification of patients with NSCLC. This registry contains free-text fields (i.e., pathological diagnosis) and *systematized nomenclature of human and veterinary medicine* (SNOMED) codes concerning pathological evaluation reports coming from regional hospitals. Moreover, data from three different registries were used to retrieve information on drug treatments: (1) the *Outpatient Drug Dispensings* (ODD) registry, which records the specific medicinal product used during outpatient encounters or dispensed for out-of-hospital use (high-cost drugs used during hospitalization are in some cases recorded, although it rarely occurs); (2) the registries of *Hospital Discharge Records* (HDR); and (3) the *Outpatient Encounter and diagnostic procedures Data* (OED), in which infusive antineoplastic administration procedures executed during hospitalization and outpatients encounters, respectively, are recorded as either unspecified standard chemotherapy or monoclonal antibody therapy.

### 2.2. Cohort Selection

NSCLC cases recorded between January 2009 and June 2019 were extracted from the regional pathology registry through the application of an identification algorithm developed ad hoc for the objectives of the study. As described elsewhere, the algorithm was based on the selection of records containing SNOMED codes and keywords used within the free-text description of the pathological diagnosis [11]. The date of the first record in the pathology registry was considered the cohort entry date. To identify patients with a primary cancer diagnosis, those with a record of non-pulmonary cancer diagnosis identified in AHD during the five years prior to the cohort entry date were excluded. Underage patients and those with less than two years of look-back in AHD were also excluded. Finally, to select incident cases of primary, non-resectable NSCLC cases, only patients without a lung cancer diagnosis recorded in AHD from three months to five years prior cohort entry and without a record of lung surgery during six months before or after the cohort entry date were retained in the study cohort. The exclusion of patients with a lung surgery record was done in order to select only patients with an advanced/metastatic stage NSCLC, as previously done in other observational studies using administrative data [12,13,14]. The selected cases were subsequently stratified in three sub-cohorts based on NSCLC histology: squamous, non-squamous, or unknown histology.

### 2.3. First-Line Identification

The pharmacological treatment of cancer is rarely represented by the administration of a single anticancer drug, but more often, it is based on the administration of more than one antineoplastic drug administered during a time frame of theoretically maximum 21 days (i.e., one treatment cycle). For each patient in the study cohort, the first record of dispensing/administration of any antineoplastic medication observed in ODD, HDR, and OED during the four months following the cohort entry date was considered the start date of the first treatment cycle. All subsequent records of drug treatment that occurred in the 21 days following the date of initiation of the treatment cycle were identified. The observed treatment sequences were then grouped in four main treatment categories: standard chemotherapy, target therapy, immunotherapy, and therapy based on unspecified monoclonal antibodies (obtained from records of drug administration procedures identified in HDR and OED specific codes).

Patients who had no drug treatment registered within the 4 months following cohort entry were classified as untreated unless a record of radiotherapy was found in HDR and OED.

### 2.4. Survival

Overall survival (OS) of the selected cohort of patients with NSCLC was described. In particular, the survival of patients receiving any first-line pharmacotherapy was observed based on the year of cohort entry, and the study cohort was categorized as follows: 2009–2011, 2012–2014, 2015–2017, and 2018–2019.

Survival time of patients receiving a first-line pharmacotherapy was defined as the time between a patient’s date of first pharmacotherapy and death. A Kaplan–Meier curve was plotted, and the analysis was stratified on patients’ histology characteristics (squamous and non-squamous) and gender (male and female). Additionally, the median OS (CI: 95%), the two-year survival and five-year survival were calculated. A multivariate Cox proportional hazards regression analysis was performed in order to analyze survival rates by cohort entry date. The analysis was adjusted by sex, age, and concomitant radiotherapy. Hazard ratios (HR) and the respective 95% confidence intervals (95% CI) were reported. A forest plot was produced.

### 2.5. Statistical Analysis

Descriptive analyses were conducted to assess demographic and clinical characteristics of selected NSCLC patients in relation to the histology of NSCLC (squamous, non-squamous, unknown). Continuous variables were described by means and standard deviation or by median and range, while categorical variables were described by patient counts and percentages. Temporal trends of the proportion of patients by first-line treatment received were evaluated using a time series model. A Mann–Kendall test was used to assess the downward or upward statistical significance of the observed trends. As for the Cox proportional hazard regression model, the assumption of proportionality of risk was checked for each covariate, and if it was not respected, a time-dependent approach was used. R software was used to perform the analyses: in particular, the following packages were utilized: “dplyr”, “ggplot2”, “tableone”, “kendall”, “survival”, “timereg”, and “forestplot”.

## 3. Results

### 3.1. Cohort Characteristics and First-Line Antineoplastic Medications Use

**Figure 1:**
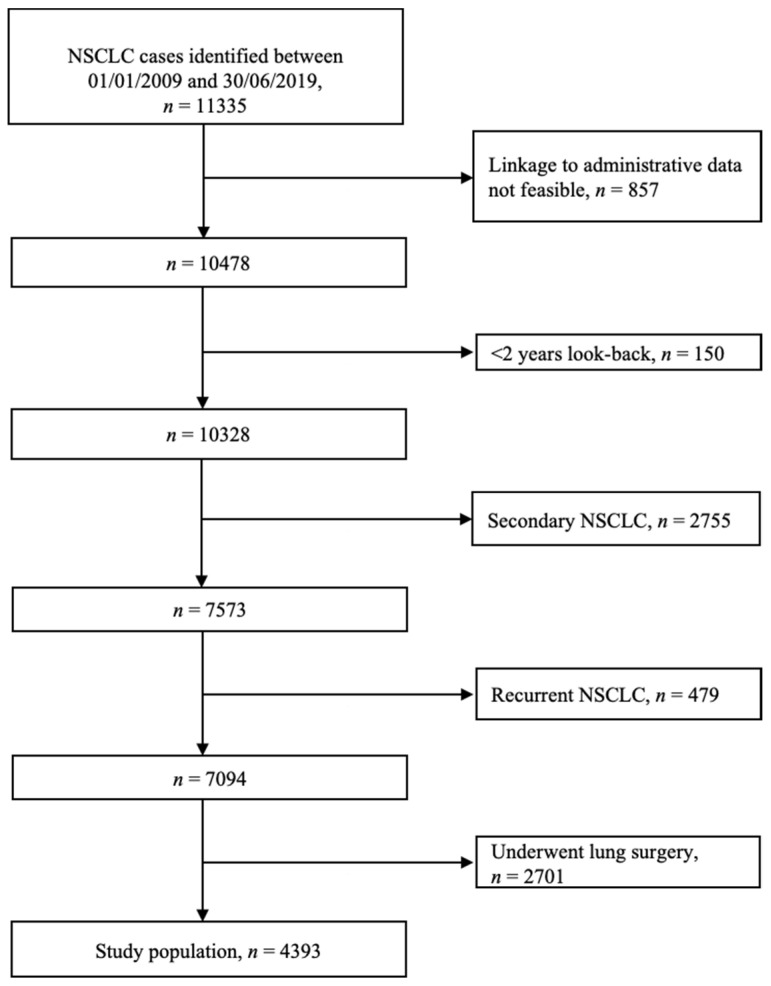
Flow chart of patient selection.

Overall, the case-finding algorithm identified 11,335 patients with NSCLC in the pathology registry of Tuscany region. Of these, a total of 4393 incident patients with unresectable primary NSCLC were included in the analysis and followed until June 2020. Cohort selection and attrition is reported in detail in Figure 1.

Within the selected cohort, 2793 patients (63.6%) had a non-squamous, 1559 (35.5%) a squamous, and 41 (0.9%) unknown histology (Table 1).

Concerning gender, female patients were more represented in non-squamous than squamous histology (37.8% vs. 18.5%). The mean age of the overall study cohort was 68.9 years, and the age band 70–84 years was the most represented for all histology and genders.

Overall, most of the patients with NSCLC had received first-line standard chemotherapy (52.6%). Approximately 10% of patients with non-squamous histology received target therapies, while these drugs were used only in few patients with squamous histology (*n* = 5). Overall, 2.5% of patients in the cohort received first-line immunotherapy. A higher proportion of patients who did not receive any treatment within four months of cohort entry was observed in patients with squamous NSCLC than patients with non-squamous NSCLC: 36.3% (female 37.0%; male 36.1%) vs. 29.0% (female 27.1%; male 30.1%). Characteristics of patients in relation to first-line treatment are available in Table S2. Concomitant radiotherapy was observed in a total of 7.6% of cases (9.1% in non-squamous and 4.9% in squamous).

**Figure 2:**
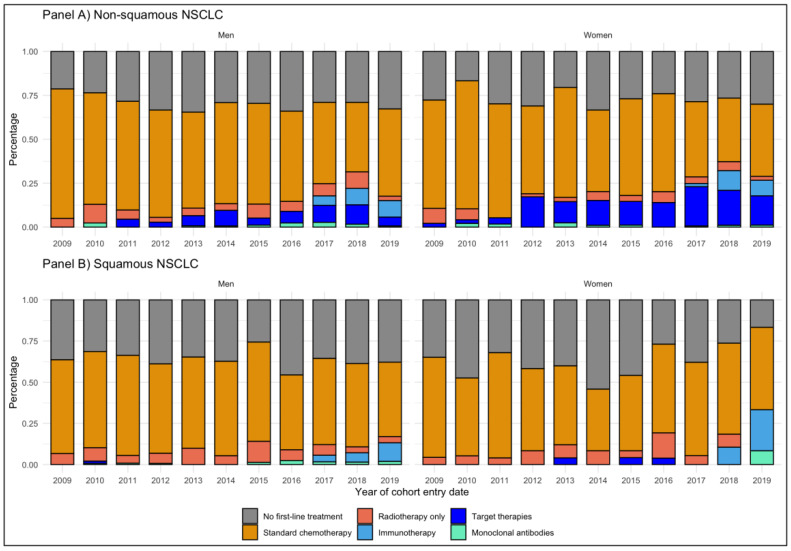
Distribution of first-line treatments per year of cohort entry: panel (**A**) non-squamous NSCLC; panel (**B**) squamous NSCLC.

The observed incidence of use of immunotherapy as first-line treatment in the study cohort varied between 3.8% in 2017 and 9.1% in 2019, without any substantial difference for histology or gender (Figure 2).

In patients with non-squamous NSCLC, first-line standard chemotherapy decreased from 2009 to 2019, both for women (from 61.7% to 41.1%) and for men (from 73.8% to 49.7%) (Figure 2, Panel A). Target therapy was used as first line for non-squamous NSCLC more frequently in women than in men (14.9% vs. 6.5%). First-line immunotherapy for non-squamous NSCLC was observed in both sexes: from 5.4% in 2017 to 9.4% in 2019 for men and from 1.9% in 2017 to 8.9% in 2019 for women. In patients with squamous NSCLC, first-line standard chemotherapy also decreased from 2009 to 2019, both for women (60.9% to 50.0%) and for men (57.0% to 45.3%) (Figure 1, Panel B). The use of target therapy was negligible in both sexes, while an increase in the use of first-line immunotherapy for squamous NSCLC was observed in both sexes: from 4.0% in 2017 to 11.3% in 2019 for men and from 0.0% in 2017 to 25.0% in 2019 for women.

The time series analysis showed a significant downward trend of the proportion of males with non-squamous and squamous NSCLC receiving standard chemotherapy (tau = −0.546, *p* value < 0.001; tau = −0.224, *p* value = 0.040, respectively). Additionally, females with non-squamous NSCLC receiving standard chemotherapy had a significant downward trend (tau = −0.495, *p* value < 0.001) (Figure S1). The time series analysis for target and immuno-therapies was not performed due to the low incidence patients treated with those two categories.

**Figure 3:**
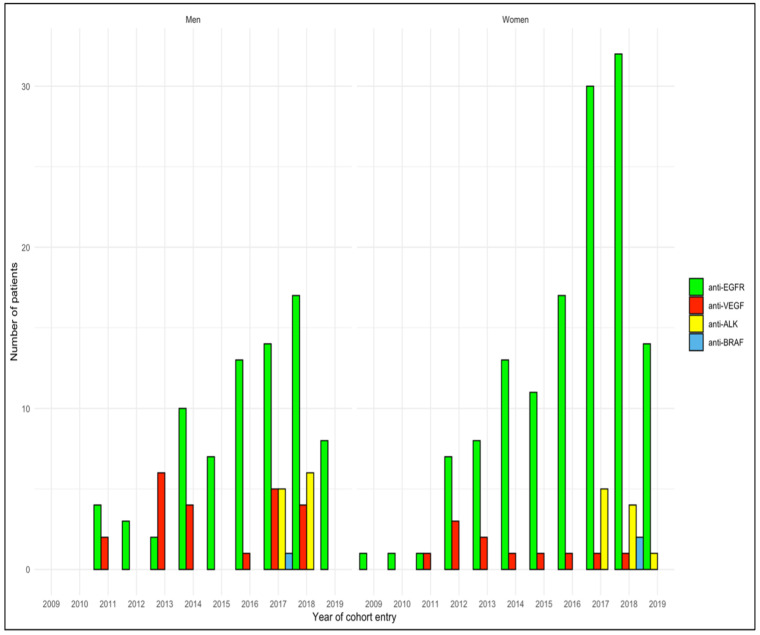
Distribution of first-line target therapies utilization over the study period among women and men with non-squamous NSCLC.

A higher utilization of anti-*EGFR* therapies in women than men out of the total number of patients treated with target therapies (85.4% vs. 69.6%) was observed (Figure 3).

Conversely, men receiving treatment with anti-Vascular endothelial growth factor (*VEGF*) and anti-*ALK* was higher than the percentage of women (19.6% vs. 7.0%, and 9.8% vs. 6.3%, respectively). Finally, only a small number of patients had received anti-*BRAF* drugs (approximately 1% for both sexes).

### 3.2. Survival

Concerning non-squamous NSCLC, among patients that received a pharmacological treatment, the median survival was 389 days in women and 276 days in men.

**Figure 4:**
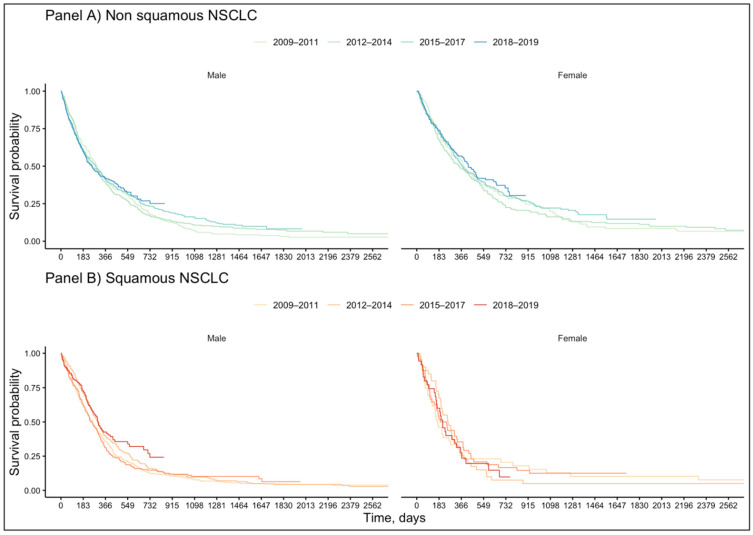
Gender-related survival of patients with non-squamous and squamous NSCLC treated with first-line pharmacotherapy. Panel (**A**): non-squamous NSCLC patients; panel (**B**) squamous NSCLC.

The two-year survival showed a trend of an increase over time: in 2009–2011, it was 30.4% for women and 17.7% for men, while it was 35.5% for women and 26.9% for men in 2018–2019. Additionally, the five-year survival showed a trend of an increase over time: in 2009–2011, it was 8.5% for women and 3.2% for men, while in 2015–2017, it was 14.7% for women and 8.3% for men (Figure 4, Panel A).

Concerning squamous NSCLC, among patients that received a pharmacological treatment, the median survival was 210 days in women and 274 days in men, without any meaningful trend over the years.

The two-year survival showed a trend to an increase over time for men: in 2009–2011, it was 12.3%, while it was 26.9% in 2018–2019. Additionally, the five-year survival showed a trend to an increase over time in men: in 2009–2011, it was 4.7%, while it was 6.4% in 2015–2017 (Figure 4, Panel B). Among women with squamous NSCLC, the estimate of five-year survival in 2015–2017 (*n* = 0) and of two-year survival in 2018–2019 (*n* = 1) was not possible due to sample size.

Characteristics of treated patients by histology and gender are presented in Table S3. While squamous patients’ characteristics for men and women are comparable, for non-squamous ones, men were slightly older than females (68 vs. 66 years).

**Figure 5:**
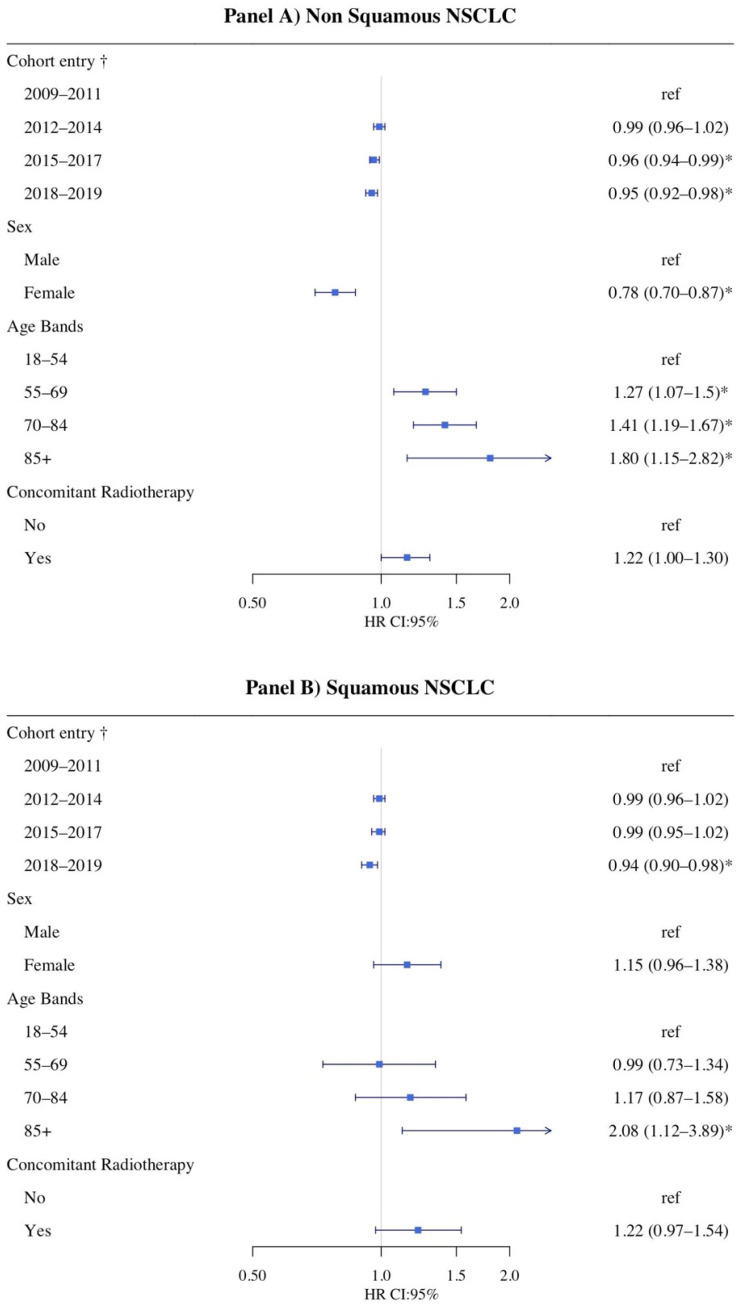
Multivariate cox proportional hazard models for survival; panel (**A**): non-squamous patients; panel (**B**): squamous patients; * *p* < 0.05; † time-dependent covariates.

Finally, an adjusted multivariate COX analysis was conducted in patients receiving a first-line treatment and reported in Figure 5.

In non-squamous patients (Figure 5, Panel A), patients that received first-line treatment in recent years (2018–2019) had slightly lower mortality rates than those who receive the treatment in 2009–2011 (HR 0.95; 95% CI: 0.92–0.98; *p* < 0.01), and women had lower mortality rates than men (*p* < 0.001).

Additionally, in squamous ones (Figure 5, Panel B), patients that received the first-line treatment in recent years (2018–2019) had slightly lower mortality rates than those who receive the treatment in 2008–2011 (HR 0.94; 95% CI: 0.90–0.98; *p* = 0.01). Conversely, women had different mortality rates with respect to those diagnosed with a non-squamous NSCLC when compared to men (HR 1.15; 95% CI: 0.96–1.38).

In both histologies, older age was associated with higher mortality. Concomitant radiotherapy was not associated with statistical difference in mortality rates (non-squamous NSCLC: *p* = 0.05; squamous *p* = 0.2).

## 4. Discussion

With this study, we described the first-line treatment utilization and survival in NSCLC patients from a whole Italian region between 2009 and 2019. During the study period, we observed a trend of decrease in the percentage of patients treated with chemotherapy. The highest percentage of cases treated with first-line target therapy was observed among women with non-squamous NSCLC, in which anti-*EGFR* medications were the most frequently used target drugs. At the same time, the survival of patients in the cohort seemed to slightly improve over the study period, especially among women with non-squamous NSCLC, although poor reduction in mortality risk was observed.

This study also improves knowledge of the re-use of data from the pathology registry of the regional AHD of Tuscany that, to the best of our knowledge, was never used before for pharmacoepidemiology purposes. The use of pathology registry allowed us to identify patients with NSCLC, also providing information on the specific cancer histology. The characteristics of the included patients are in line with epidemiological data [2]: as expected, non-squamous histology was more frequent than squamous histology on the total of NSCLC cases (70% vs. 30%), and the proportion of women with non-squamous histology was higher than the proportion of those with squamous NSCLC [15].

Concerning drug utilization, this study found that, among patients with non-squamous NSLCL, women received first-line target therapy more than twice as frequently as men (14.9% vs. 6.5%). These data are in line with the frequency of lung cancer mutations in the Caucasian population, where *EGFR* and *BRAF* mutations are known to be more frequent in women [16,17,18].

During the study period, the proportion of patients who did not receive any drug treatment in the four months following cohort entry was stable and concerned about one-third of patients with NSCLC, whatever the histology (36% squamous vs. 29% non-squamous). A previous study using the Iowa Cancer Registry reported that more than 20% of patients with advanced NSCLC were untreated, and that this percentage was higher than that observed for any other cancer, with exception of non-Hodgkin lymphoma [19]. Additionally, a National Cancer Data Base study showed comparable rates from 1998 to 2012: among patients with stage IIIB and IV NSCLC, 22.3% and 25.9% of patients did not receive any anticancer treatment, respectively [20]. The high rate of untreated patients is mainly due to the advanced stage of the disease and the often poor performance status of patients for which the small benefits expected from the treatment and their potential harms can lead physicians and patients to avoid any anticancer treatment [21].

According to the guidelines for the treatment of NSCLC, identification of activating mutations of *EGFR* and *ALK* is recommended [2]. According to the literature, 10–13% of Caucasian patients with adenocarcinoma (which represents the most part of non-squamous NSCLC) presents an *EGFR* activating mutation, 2–7% rearrangements of *ALK* and 1–4% *BRAF* mutations [2,22]. In line with these data, about 10% of patients with non-squamous NSCLC in our study cohort were treated with target therapies. Moreover, as expected from the existing differences between sexes in the expression of *EGFR* activating mutation [13,14], women with non-squamous NSCLC in the present study cohort received a first-line target therapy more than twice as frequently as men with non-squamous NSLCL.

During the study period, immunotherapy (e.g., pembrolizumab) was approved as a first-line treatment for NSCLC by FDA and EMA. In non-squamous NSCLC, pembrolizumab is reserved for patients not eligible for target therapy, in combination with chemotherapy or as monotherapy, but only in patients with >50% of PD-L1 mutation. It could be conversely used in combination with standard chemotherapy in the squamous ones. Other observational studies that focused on the use of first-line pembrolizumab in NSCLC patients [23,24,25] did not analyze the incidence of use of pembrolizumab in patients with advanced NSCLC. Thus, to our knowledge, the present study estimated for the first time the incidence of use of first-line immunotherapy in non-resectable NSCLC cancer after approval and showed an increase in its use in both squamous and non-squamous histologies in both sexes.

Concerning survival, the present study seemed to show a slight overall trend of improvement of patients’ prognosis over time, which may be in part related to the marketing of novel anticancer drugs. In the Kaplan–Meier analysis (Figure 4), independently of NSCLC histology and with no specific difference in the characteristics of treated patients between male and female (Table S3), OS seemed to slightly increase over time both in terms of two- and five-year survival. Only in squamous female patients was this trend not observed. Additionally, few women with squamous histology were included in this study, and no conclusion can be drawn for this subgroup. Nevertheless, after a COX multivariate analysis (Figure 5), the mortality risk was only slightly reduced for those patients receiving a first-line treatment in the period 2018–2019 with respect to those receiving a treatment in 2009–2011. Thus, although this result was found to be statistically significant in both squamous and non-squamous patients, from a clinical point of view, the reduction in mortality risk is still too poor in these patients.

In the present study, among patients with non-squamous NSCLC that received any anticancer drugs, a better OS was observed in women compared to men (HR 0.78; 95% CI: 0.70–0.87). This result could be in part related to the different biomolecular characteristics between male and female, which allowed women to be more eligible to the first line treatment with target therapies, as also confirmed by the pattern of drug utilization. For the squamous one, OS seems better in men than in women, as already suggested by other studies [26,27].

This study has several strengths. The first is represented by the linkage of different registries from the administrative healthcare database of Tuscany, which provided detailed information at patient level, treatment information, and cancer histology. The second strength is represented by the use of an ad hoc case-finding algorithm developed and validated for the purpose of this study. The algorithm was shown to have a high PPV (93%) [11]. Finally, to the best of our knowledge, this is the first population-based observational study that estimated the incidence of use of immunotherapy as a first-line treatment in patients with non-resectable NSCLC in real-world clinical practice in Europe.

The study also has some limitations. The first concerns the sensitivity of the algorithm used for the extraction of NSCLC cases. Although it demonstrated a very high PPV, the application of the algorithm to the regional pathology registry was found to be able to identify about the half of the cases of NSCLC that occurred in the Tuscan population [11]. However, the distribution of patient characteristics (i.e., histological types) in the present study cohort was line with the epidemiology of the disease, so that the sample of cases identified can be considered representative of the underlying source case population. Secondly, the accuracy of the identification of the first-line pharmacotherapy was not validated. Given the universal healthcare assistance granted to all Italian inhabitants and the extremely high costs associated with NSCLC treatment, the possible impact of private healthcare assistance (which is not tracked in the administrative data used for this study) on exposure misclassification is expected to be negligible. Nevertheless, in some cases, we cannot exclude that the date of actual administration of drugs might, in some cases, differ from the date registered in the administrative databases used for this study. Thirdly, two of the databanks used for the identification of drug exposure (HDR and OED) only allowed us to identify “unspecified anti-body therapy”, which could correspond either to an immunotherapy or a bevacizumab-based target therapy. Nevertheless, patients treated with drugs classified as “unspecified anti-body therapy” represents only 1.0% of the entire cohort. Finally, performance status could not be retrieved in the administrative database of the Tuscany region. It is well known that performance status is very poor in the most part of patients that did not receive any treatment, even if a small part of them could be eligible but refuse to receive treatment [28,29]. The time series analysis showed that the percentage of non-treated patients remained stable over the years, suggesting that the performance status of included patients is stable over the study period. So, it is unlikely that this information could have markedly influenced our results; nevertheless, as the observed difference in survival over the study period is small, we cannot exclude that performance status could have an impact on our results.

## 5. Conclusions

In conclusion, this study demonstrated that Italian administrative healthcare data can be a valuable tool for the study of drug utilization and survival in oncology field. In particular, this study described, over last decade, the introduction of target and immunotherapies for the treatment of patients with non-resectable NSCLC in clinical practice. Our findings highlight a different use of target therapies between sexes: in women with non-squamous NSCLC, anti-*EGFR* medications were the most frequently used target drugs according to the known biomolecular profile of females. In patients receiving a first-line treatment, survival seemed to slightly improve over the study period for both histologies, although a still poor reduction in mortality risk was observed. Male and female patients also presented a different survival profile if diagnosed with a squamous or a non-squamous NSCLC.

Future studies on this topic will be able to leverage evidence and methodologies from this work to provide a more detailed picture of NSCLC pharmacotherapy utilization (i.e., second-line treatment), safety, and effectiveness in real-life, with a special focus on innovative medications and gender.

## Figures and Tables

**Table 1:** Cohort characteristics by histology and sex.

Patients’ Characteristics	Non-Squamous		Squamous		Unknown Histology		Overall *n* = 4393 (%)
	*n* = 2793		*n* = 1559		*n* = 41		
	Women *n* = 1057 (%)	Men *n* = 1736 (%)	Women *n* = 289 (%)	Men *n* = 1270 (%)	Women *n* = 13 (%)	Men *n* = 28 (%)	
Age at cohort entry date, mean (SD)	67.5 (11.5)	69.6 (9.7)	70.7(9.0)	71.8 (9.2)	68.9 (11.1)	70.6 (10.7)	69.8 (10.1)
Age bands at cohort entry date							
18–54	144 (13.6)	136 (7.8)	17 (5.9)	67 (5.3)	2 (15.4)	4 (14.3)	370 (8.4)
55–69	424 (40.1)	671 (38.7)	100 (34.6)	395 (31.1)	3 (23.1)	6 (21.4)	1599 (36.4)
70–84	434 (41.1)	856 (49.3)	163 (56.4)	732 (57.6)	8 (61.5)	15 (53.6)	2208 (50.3)
85+	55 (5.2)	73 (4.2)	9 (3.1)	76 (6.0)	0	3 (10.7)	216 (4.9)
Time in the study cohort, median (IQR)	338.0 (113, 685)	211.0 (72, 533)	201.0 (70, 424)	228.0 (78, 444)	400.0 (140, 750)	134.0 (50, 370)	239.0 (80, 540)
First-line pharmacotherapies							
Standard chemotherapy	526 (49.8)	921 (53.1)	151 (52.2)	694 (54.6)	6 (46.2)	13 (46.4)	2311 (52.6)
Target therapies	158 (14.9)	112 (6.5)	3 (1.0)	2 (0.2)	4 (30.8)	0 (0.0)	279 (6.4)
Immunotherapy	33 (3.1)	52 (3.0)	7 (2.4)	19 (1.5)	0 (0.0)	0 (0.0)	111 (2.5)
Unspecified anti-body therapy	10 (0.9)	23 (1.3)	1 (0.3)	12 (0.9)	0 (0.0)	0 (0.0)	46 (1.0)
Radiotherapy only	44 (4.2)	105 (6.0)	20 (6.9)	84 (6.6)	1 (7.7)	2 (7.1)	256 (5.8)
Untreated	286 (27.1)	523 (30.1)	107 (37.0)	459 (36.1)	2 (15.4)	13 (46.4)	1390 (31.6)
Concomitant radiotherapy	103 (9.7)	151 (8.7)	18 (6.2)	59 (4.6)	2 (15.4)	0	333 (7.6)
Diagnostic imaging							
Computed tomography	745 (70.5)	1280 (73.7)	215 (74.4)	973 (76.6)	10 (76.9)	15 (53.6)	3238 (73.7)
Radiography	509 (48.2)	875 (50.4)	154 (53.3)	726 (57.2)	7 (53.8)	21 (75.0)	2292 (52.2)

SD: standard deviation; IQR: interquartile range.

## Data Availability

The data presented in this study are available in the article and Supplementary Materials.

## Supplementary Materials

The following are available online at https://www.mdpi.com/article/10.3390/cancers13236129/s1, Figure S1: Times series analysis: percentage of untreated patients and of those receiving standard chemotherapy during the study period per calendar quarters, Table S1: Target and immuno-therapies approved for the treatment of advanced/metastatic stage NSCLC during study period, Table S2: Characteristics of patients on the basis of first line received, Table S3: Characteristics of treated patients by sex.
