# Supplementary material for: First-Line Pharmacotherapies and Survival among Patients Diagnosed with Non-Resectable NSCLC: A Real-Life Setting Study with Gender Prospective
Source: Cancers (Basel). 2021 Dec 5;13(23):6129. doi: 10.3390/cancers13236129 (PMC8657179; doi:10.3390/cancers13236129)
Supplement: Supplementary file 1 [file cancers-13-06129-s001.zip › cancers-1443996-supplementary.pdf]

Supplementary Materials

Figure S1. Times series analysis: percentage of untreated patients and of those receiving standard chemotherapy during the study period per calendar quarters. Panel (A1) Non-squamous NSCLC, Male; Panel (A2) Non-squamous NSCLC, Female; Panel (B1) Squamous NSCLC, Male; Panel (B2) Squamous NSCLC, Female

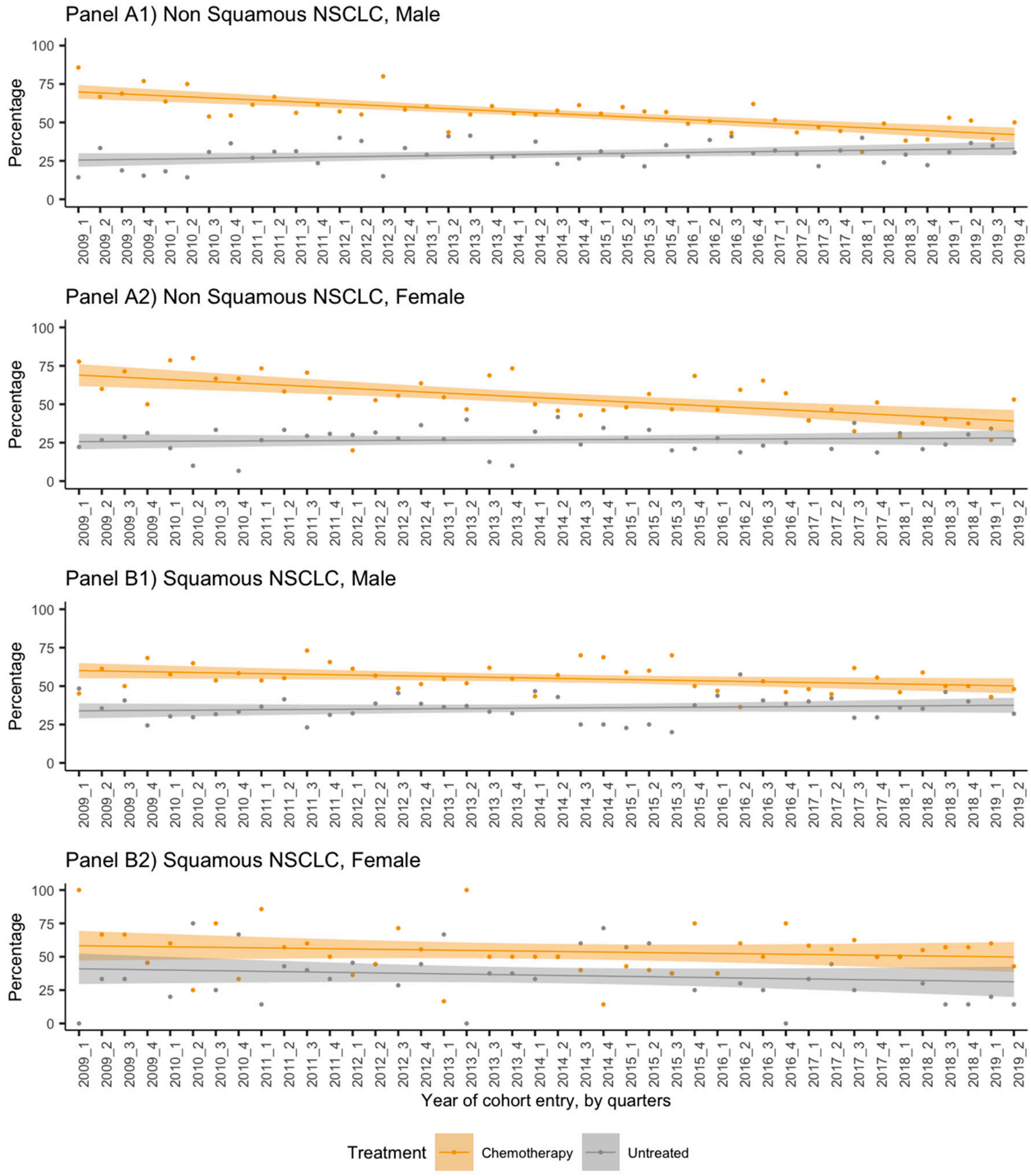

	Non squamous NSCLC				Squamous NSCLC			
	**Male**		**Female**		**Male**		**Female**	
	**Chemotherapy**	**Untreated**	**Chemotherapy**	**Untreated**	**Chemotherapy**	**Untreated**	**Chemotherapy**	**Untreated**
Trend	tau = -0.546	tau = 0.136	tau = -0.495	tau= 0.0269	tau = -0.224	tau = 0.119	tau = -0.0414	tau = -0.0962
P value	**5.4319e-07***	0.21655	**5.6485e-06***	0.81346	**0.040784***	0.28062	0.7178	0.3917

*Statistically significant

Table S1. Target- and immuno-therapies approved for the treatment of advanced/metastatic stage NSCLC

Drugs	Target	Approval date (EMA)
**Target therapy**		
Erlotinib	EGFR	3 November 2005, second-line<br>14 November 2011, first-line
Gefitinib	EGFR	24 June 2009
Afatinib	EGFR	25 September 2013
Osimertinib	EGFR	02 February 2016
Dacomitinib	EGFR	25 June 2019
Crizotinib	ALK	23 October 2012
Ceritinib	ALK	06 May 2015
Alectinib	ALK	16 February 2017
Brigatinib	ALK	22 November 2018
Lorlatinib	ALK	17 June 2019
Dabrafenib	BRAF	29 March 2017
Trametinib	BRAF	27 March 2017
Bevacizumab	VEGF	21 August 2007
Nintedanib	VEGFR, PDGFR	08 January 2015
Ramucirumab	VEGF	25 January 2016
**Immunotherapy**		
Nivolumab	PD-1	
Pembrolizumab	PD-1	17 July 2015
Atezolizumab	PD-L1	29 September 2017
Durvalumab	PD-L1	21 September 2018
Ipilimumab	Anti-CTLA	17 September 2020

Table S2. Characteristics of patients on the basis of first-line received

	Non squamous						Squamous					
	**CT**	**TT**	**IT**	**uMAB**	**RT**	**NT**	**CT**	**TT**	**IT**	**uMAB**	**RT**	**NT**
n	1447	270	85	33	149	809	845	5	26	13	104	566
Female (%)	526 (36.4)	158 (58.5)	33 (38.8)	10 (30.3)	44 (29.5)	286 (35.4)	151 (17.9)	3 (60.0)	7 (26.9)	1 (7.7)	20 (19.2)	107 (18.9)
YearCohortEntryDate (%)												
2009	74 (5.1)	1 (0.4)	0 (0.0)	0 (0.0)	7 (4.7)	26 (3.2)	91 (10.8)	0 (0.0)	0 (0.0)	0 (0.0)	10 (9.6)	57 (10.1)
2010	89 (6.2)	1 (0.4)	0 (0.0)	3 (9.1)	12 (8.1)	28 (3.5)	95 (11.2)	2 (40.0)	0 (0.0)	1 (7.7)	13 (12.5)	55 (9.7)
2011	120 (8.3)	8 (3.0)	0 (0.0)	1 (3.0)	7 (4.7)	55 (6.8)	94 (11.1)	0 (0.0)	0 (0.0)	1 (7.7)	7 (6.7)	51 (9.0)
2012	95 (6.6)	13 (4.8)	0 (0.0)	0 (0.0)	4 (2.7)	54 (6.7)	98 (11.6)	0 (0.0)	0 (0.0)	1 (7.7)	12 (11.5)	72 (12.7)
2013	128 (8.8)	18 (6.7)	0 (0.0)	3 (9.1)	8 (5.4)	65 (8.0)	68 (8.0)	1 (20.0)	0 (0.0)	0 (0.0)	12 (11.5)	45 (8.0)
2014	137 (9.5)	28 (10.4)	0 (0.0)	2 (6.1)	11 (7.4)	79 (9.8)	63 (7.5)	0 (0.0)	0 (0.0)	0 (0.0)	7 (6.7)	48 (8.5)
2015	150 (10.4)	19 (7.0)	0 (0.0)	3 (9.1)	17 (11.4)	76 (9.4)	58 (6.9)	1 (20.0)	0 (0.0)	1 (7.7)	11 (10.6)	31 (5.5)
2016	181 (12.5)	32 (11.9)	0 (0.0)	5 (15.2)	20 (13.4)	103 (12.7)	70 (8.3)	1 (20.0)	0 (0.0)	3 (23.1)	12 (11.5)	63 (11.1)
2017	189 (13.1)	61 (22.6)	17 (20.0)	8 (24.2)	24 (16.1)	121 (15.0)	86 (10.2)	0 (0.0)	5 (19.2)	2 (15.4)	10 (9.6)	58 (10.2)
2018	168 (11.6)	66 (24.4)	45 (52.9)	6 (18.2)	33 (22.1)	123 (15.2)	92 (10.9)	0 (0.0)	12 (46.2)	2 (15.4)	8 (7.7)	64 (11.3)
2019	116 (8.0)	23 (8.5)	23 (27.1)	2 (6.1)	6 (4.0)	79 (9.8)	30 (3.6)	0 (0.0)	9 (34.6)	2 (15.4)	2 (1.9)	22 (3.9)
AgeAtCohortEntryDate (mean (SD))	66.38 (9.83)	68.51 (10.94)	66.89 (10.31)	67.94 (8.94)	72.93 (9.14)	72.82 (10.42)	69.12 (8.76)	70.00 (5.52)	71.04 (8.77)	68.77 (8.86)	75.72 (7.68)	74.64 (8.93)
AgeBandAtCohortEntryDate (%)												
18-54	183 (12.6)	32 (11.9)	12 (14.1)	2 (6.1)	8 (5.4)	43 (5.3)	57 (6.7)	0 (0.0)	0 (0.0)	1 (7.7)	1 (1.0)	25 (4.4)
55-69	676 (46.7)	101 (37.4)	35 (41.2)	15 (45.5)	38 (25.5)	230 (28.4)	344 (40.7)	3 (60.0)	11 (42.3)	5 (38.5)	20 (19.2)	112 (19.8)
70-84	573 (39.6)	127 (47.0)	36 (42.4)	16 (48.5)	92 (61.7)	446 (55.1)	434 (51.4)	2 (40.0)	12 (46.2)	7 (53.8)	75 (72.1)	365 (64.5)
85+	15 (1.0)	10 (3.7)	2 (2.4)	0 (0.0)	11 (7.4)	90 (11.1)	10 (1.2)	0 (0.0)	3 (11.5)	0 (0.0)	8 (7.7)	64 (11.3)
LengthObservationPeriod (median [IQR])	322.00 [154.00, 615.50]	538.00 [255.25, 793.50]	438.00 [157.00, 575.00]	374.00 [147.00, 635.00]	154.00 [58.00, 609.00]	62.00 [26.00, 294.00]	301.00 [157.00, 496.00]	199.00 [105.00, 645.00]	344.50 [115.25, 527.50]	232.00 [171.00, 453.00]	178.50 [69.00, 694.25]	77.50 [32.00, 275.50]
TC Chest = Yes (%)	1095 (75.7)	191 (70.7)	62 (72.9)	16 (48.5)	114 (76.5)	547 (67.6)	657 (77.8)	4 (80.0)	18 (69.2)	8 (61.5)	83 (79.8)	418 (73.9)
RX Chest = Yes (%)	783 (54.1)	119 (44.1)	32 (37.6)	16 (48.5)	66 (44.3)	368 (45.5)	501 (59.3)	3 (60.0)	15 (57.7)	6 (46.2)	43 (41.3)	312 (55.1)

CT: Standard chemotherapy; TT: Target therapies; IT: Immunotherapy; uMAB: Unspecified monoclonal antibodies; RT: Radiotherapy; NT: Untreated

Table S3. Characteristics of treated patients by sex

	Non squamous		Squamous	
	**Male**	**Female**	**Male**	**Female**
n	1213	771	811	182
YearCohortEntryDate (%)				
2009	48 (4.0)	34 (4.4)	86 (10.6)	15 (8.2)
2010	65 (5.4)	40 (5.2)	101 (12.5)	10 (5.5)
2011	96 (7.9)	40 (5.2)	85 (10.5)	17 (9.3)
2012	72 (5.9)	40 (5.2)	90 (11.1)	21 (11.5)
2013	91 (7.5)	66 (8.6)	66 (8.1)	15 (8.2)
2014	112 (9.2)	66 (8.6)	59 (7.3)	11 (6.0)
2015	124 (10.2)	65 (8.4)	58 (7.2)	13 (7.1)
2016	140 (11.5)	98 (12.7)	67 (8.3)	19 (10.4)
2017	184 (15.2)	115 (14.9)	80 (9.9)	23 (12.6)
2018	174 (14.3)	144 (18.7)	86 (10.6)	28 (15.4)
2019	107 (8.8)	63 (8.2)	33 (4.1)	10 (5.5)
AgeAtCohortEntryDate (mean (SD))	68.07 (9.36)	65.85 (11.03)	70.00 (8.80)	69.26 (9.17)
AgeBandAtCohortEntryDate (%)				
18-54	114 (9.4)	123 (16.0)	47 (5.8)	12 (6.6)
55-69	532 (43.9)	333 (43.2)	305 (37.6)	78 (42.9)
70-84	542 (44.7)	302 (39.2)	440 (54.3)	90 (49.5)
85+	25 (2.1)	13 (1.7)	19 (2.3)	2 (1.1)
LengthObservationPeriod (median [IQR])	298.00 [138.00, 606.00]	399.00 [189.50, 729.50]	307.00 [147.00, 533.00]	244.50 [131.50, 433.50]
